# Medical disciplinary jurisprudence in alleged malpractice in radiology: 10-year Dutch experience

**DOI:** 10.1007/s00330-020-06685-0

**Published:** 2020-02-17

**Authors:** Robert M. Kwee, Thomas C. Kwee

**Affiliations:** 1 Department of Radiology, Zuyderland Medical Center, Heerlen/Sittard/Geleen, The Netherlands; 2 Department of Radiology, Nuclear Medicine and Molecular Imaging, University Medical Center Groningen, University of Groningen, Hanzeplein 1, P.O. Box 30.001, 9700 RB Groningen, The Netherlands

**Keywords:** Medical errors, Diagnostic errors, Malpractice, Radiology, Jurisprudence

## Abstract

**Purpose:**

To systematically investigate the frequency and types of allegations related to radiology practice handled by the Dutch Medical Disciplinary Court in the past 10 years.

**Methods:**

The Dutch Medical Disciplinary Court database was searched for verdicts concerning radiology practice between 2010 and 2019. The association between the number of verdicts and time (years) was assessed by Spearman’s rho. Other data were summarized using descriptive statistics.

**Results:**

There were 48 verdicts (mean 4.8 per year). There was no significant association between the number of verdicts and time (Spearman’s rho < 0.001, *p* = 0.99). Most allegations were in breast imaging and musculoskeletal radiology (each 18.8%), followed by interventional radiology, head and neck imaging, and abdominal imaging (each 12.5%), neuroradiology and vascular imaging (each 10.4%), and chest imaging (4.2%). There were 46 allegations against radiologists (95.8%) and 2 against residents (4.2%). The most common allegation (37.5%) was error in diagnosis. In 20.8% of verdicts, the allegation was judged (partially) founded; disciplinary measures were warnings (*n* = 8) and reprimands (*n* = 2). An appeal was submitted by the patient in 11 cases and by the radiologist in 3 cases. All appeals by patients were rejected, whereas 2 of the 3 appeals by radiologists were granted and previously imposed disciplinary measures were reversed.

**Conclusion:**

Allegations against radiologists at the Dutch Medical Disciplinary Court are relatively few, their number has remained stable over the past 10 years, and a minority were judged to be (partially) founded. We can learn from the cases presented in this article, which may improve patient care.

**Key Points:**

*• The frequency of allegations against radiologists at the Dutch Medical Disciplinary Court is relatively low and has not exhibited any temporal change over the past 10 years.*

*• These allegations reflect patient dissatisfaction, but this infrequently equals malpractice.*

*• Knowledge of the circumstances under which these allegations have arisen may improve patient care.*

## Introduction

Radiology is one of the medical specialties with the highest number of malpractice suits in the USA [1]. The likelihood of a radiologist in the USA being the defendant in at least one suit is 50% by age 60 [2]. It can be expected that the number of malpractice suits will further increase [3, 4]. Data from the USA show that diagnostic errors are by far the most common cause of malpractice suits, whereas failure to communicate and failure to recommend additional testing are both uncommon reasons for initiating a suit [1, 5]. There are relatively few published data regarding malpractice suits against radiologists in Europe [6, 7] compared with those against radiologists in the USA [1, 2, 5, 8–17]. The medical disciplinary law system in The Netherlands is unique and essentially different from a medical malpractice claim system, because its main objective is to maintain and improve the quality of healthcare rather than punishing healthcare professionals and/or financially compensating patients [18, 19]. In addition, patients can allege healthcare professionals without proceeding to civil court or insurance claims. To our knowledge, verdicts by the Dutch Medical Disciplinary Court related to radiology practice have not been systematically investigated yet. In addition, it is still unknown if the frequency of patient allegations has remained stable or if it has changed over the years. This information may be helpful to radiologists to improve the care they provide to their patients, and to prevent patient dissatisfaction and allegations. Furthermore, this experience may be valuable to governmental bodies and healthcare policy makers in other countries who wish to reform their medical disciplinary law system from a malpractice claim system into a system akin the Dutch. Therefore, the objective of our study was to systematically investigate the frequency and types of allegations related to radiology practice handled by the Dutch Medical Disciplinary Court in the past 10 years.

## Methods

The online database of the Dutch Medical Disciplinary Court is publicly available and all data are anonymized. Therefore, ethics committee approval was not applicable for this study.

### Data collection

The database of the Dutch Medical Disciplinary Court (https://tuchtrecht.overheid.nl/nieuw/gezondheidszorg) was searched for verdicts published in the past 10 years (2010–2019). All cases handled by this institute (which consists of independent medical and legal experts) are published in detail online 1 day after the verdict. Only verdicts concerning allegations against radiologists or radiology residents were selected and included in the present study. Verdicts concerning allegations which were not directly related to radiology practice (such as private affairs or non-radiological work) were excluded.

### Data extraction and analysis

The following data were extracted for each verdict: radiological subspecialty, whether a radiologist or resident was alleged, number of days between date of filing the allegation and date of the verdict, the type of allegation, the verdict, the type of disciplinary measure (Table 1), if the allegation was judged to be (partially) founded, and whether there was an appeal against the verdict. In order to determine whether the number of verdicts has either increased or remained stable over time, we calculated Spearman’s rho between the number of verdicts and time (years). Other data were summarized using descriptive statistics. In cases in which the allegation was judged (partially) founded, we determined (potential) causes that have led to error/malpractice [20, 21].

**Table 1:** Disciplinary measures which can be imposed by the Dutch Medical Disciplinary Court, in order of severity

1. Warning*	
2. Reprimand^#^	
3. Monetary fine up to a maximum of 4.500 €	
4. Suspension for a maximum of 1 year	
5. Partial prohibition to practice	
6. Total prohibition to practice	

*A warning represents the lightest measure: it is a reproof for misconduct (but not for culpable negligence) and has no direct consequences to the healthcare professional. A warning is neither published in the publicly available Dutch registry for healthcare professionals nor in a local newspaper

^#^A reprimand represents a more severe measure: it is a reproof for culpable negligence. A reprimand is published in the Dutch registry for healthcare professionals and will be available for 5 years. Furthermore, a reprimand may be published in a local newspaper, if decided upon by the Dutch Medical Disciplinary Court

## Results

**Fig. 1:**
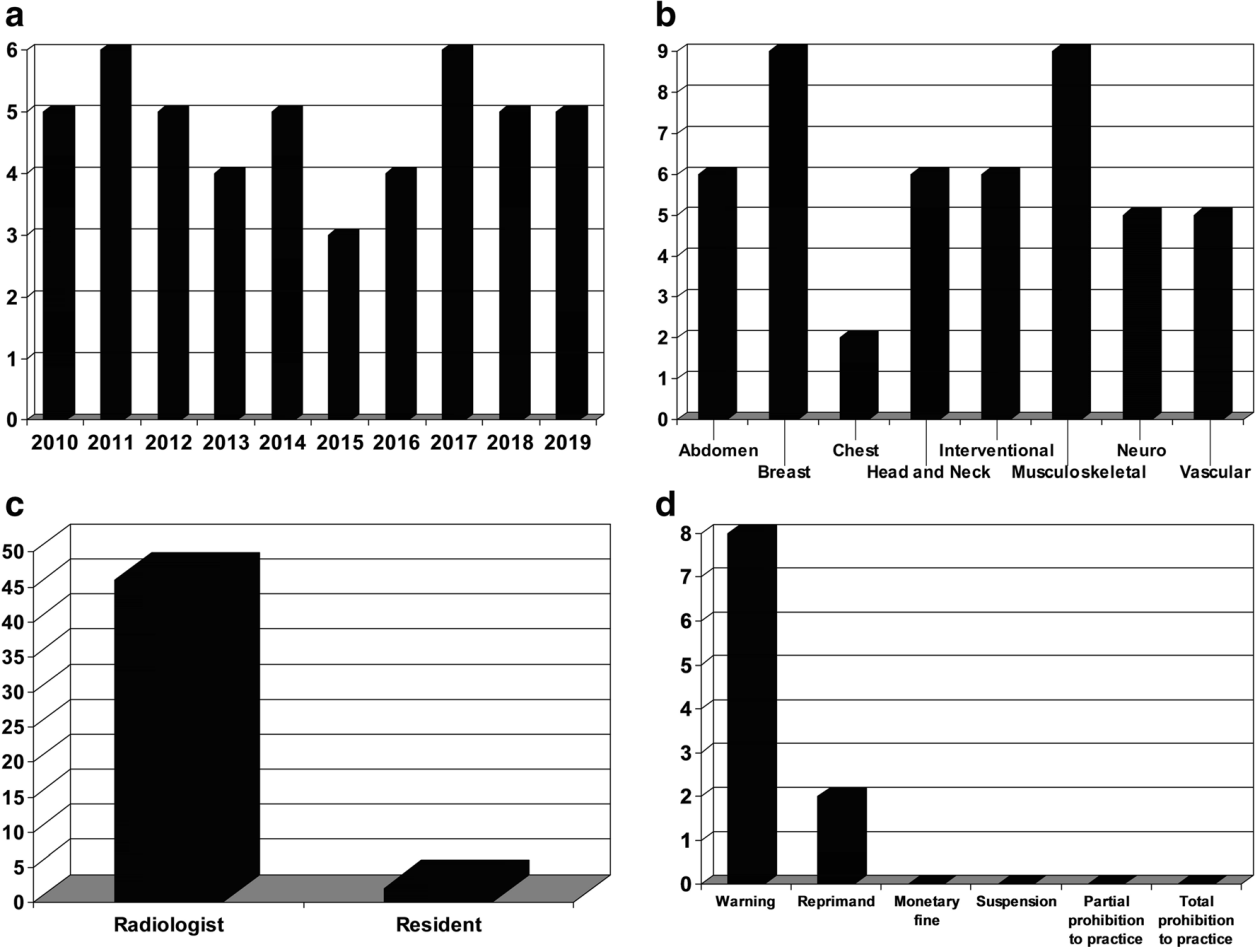
Number of verdicts by the Dutch Medical Disciplinary Court for each year between 2010 and 2019 (**a**), number of allegations per subspecialty (**b**), number of allegations against radiologists and residents (**c**), and types of disciplinary measures for the 10 verdicts in which the allegation was judged (partially) founded by the Dutch Medical Disciplinary Court (**d**)

There were 52 verdicts. Four verdicts were excluded, because they were not directly related to radiology practice. Eventually, 48 verdicts were included (Table 2).There was no significant association between the number of verdicts and time (Spearman’s rho < 0.001, *p* = 0.99) (Fig. 1a). Most allegations were in breast imaging and musculoskeletal radiology (each 18.8%), followed by interventional radiology, head and neck imaging, and abdominal imaging (each 12.5%), neuroradiology and vascular imaging (each 10.4%), and chest imaging (4.2%) (Fig. 1b). There were 46 allegations against radiologists (95.8%) and 2 allegations against residents (4.2%) (Fig. 1c). The most common allegation was error in diagnosis (19/48 cases, 39.6%). In 10/48 verdicts (20.8%), the allegation was judged (partially) founded; disciplinary measures were warnings (*n* = 8) and reprimands (*n* = 2) (Fig. 1d). All 11 appeals by patients were rejected, whereas 2 of 3 appeals by radiologists were granted and the previously imposed disciplinary measures were reversed. (Potential) causes leading to error/malpractice in cases in which the allegation was judged (partially) founded are displayed in Table 3.

**Table 2:** Summary of verdicts against radiologists by the Dutch Medical Disciplinary Court between 2010 and 2019

Case no.	Year of verdict	Subspecialty	Defendant	Days between filing allegation and verdict	Allegations^	Use of attorney by patient during court session	Use of attorney by defendant during court session	Verdict	Disciplinary measure	Appeal and result
1	2010	Vascular	Radiologist	277	Providing incorrect information to the referring physician and failure to detect the patient’s coagulation disorder	No	Yes	Unfounded		
2	2010	Breast	Radiologist	470	Error in diagnosis	No	No	Unfounded		
3	2010	Breast	Radiologist	526	Not receiving the result of breast screening mammogram	Yes	No	Unfounded		
4	2010	Interventional	Radiologist	504	Performing additional angiographic recordings and not aborting the procedure, incorrect manual compression of the arterial access site, and no show after the procedure	Yes	Yes	Unfounded		Yes, rejected
5	2010	Breast	Radiologist	489	Failure to perform mammography or to refer patient to a surgeon, *incorrect reporting that patient refused to undergo mammography, and error in diagnosis*	Yes	Yes	Partially founded	Reprimand	Yes, rejected
6	2011	Vascular	Radiologist	364	*Failure to verbally communicate emergent critical findings to the referring physician*	Yes	Yes	Founded	Reprimand	Yes, granted
7	2011	Chest	Radiologist	406	Failure to directly communicate a critical finding to the referring physician	No	Yes	Unfounded		
8	2011*	Head and neck	Radiologist	365	Failure to build a good doctor-patient relationship, refusal to discuss radiological reports with patient, and making agreements with colleagues to make an incorrect conclusion about the MRI scans	No	Yes	Unfounded		Yes, rejected
9	2011*	Head and neck	Radiologist	365	Incorrect doctor-patient interaction, incorrect use of contrast medium, adjustment of initial radiology report, failure to build a good doctor-patient relationship, refusal to discuss radiological reports, and making agreements with colleagues to make an incorrect conclusion about the MRI scans	No	Yes	Unfounded		Yes, rejected
10	2011*	Head and neck	Radiologist	365	Error in diagnosis and incorrect reporting, failure to build a good doctor-patient relationship, refusal to discuss radiological reports, and making agreements with colleagues to make an incorrect conclusion about the MRI scans	No	Yes	Unfounded		Yes, rejected
11	2011	Abdomen	Radiologist	432	Providing insufficient information about oral contrast agent, insufficient attention for patient’s allergy to iodinated contrast medium, and inappropriate action when patient felt unwell	No	No	Unfounded		Yes, rejected
12	2011	Breast	Radiologist	440	Incorrect interpretation, use of insufficient equipment, and failure to respond to the initial complaint against the radiologist at the hospital	Yes	Yes	Unfounded		
13	2012	Musculoskeletal	Radiologist	436	Error in diagnosis	Yes	Yes	Unfounded		
14	2012	Abdomen	Radiologist	370	Error in diagnosis	Yes	Yes	Unfounded		
15	2012	Abdomen	Radiologist	370	Error in diagnosis	Yes	Yes	Unfounded		
16	2012	Breast	Radiologist	259	Providing incorrect information about radiologic findings, failure to refer patient to the GP, and failure to instruct patient to return in case of growth of the breast lump	Yes	No	Unfounded		
17	2013	Breast	Resident	206	Error in diagnosis	No	Yes	Unfounded		
18	2013	Breast	Radiologist	206	*Error in diagnosis* or insufficiently defending correct radiologic findings in multidisciplinary team meeting	No	Yes	Partially founded	Warning	
19	2013	Head and neck	Radiologist	396	Refusal to give a second opinion and not referring the case to a colleague	No	Yes	Unfounded		Yes, rejected
20	2013	Abdomen	Radiologist	505	Error in diagnosis and failure to contact colleagues from another hospital	No	Yes	Unfounded		
21	2014	Neuro	Radiologist	565	Wrong body part scanned	No	Yes	Unfounded		
22	2014	Head and neck	Radiologist	344	*Error in diagnosis* and failure to have a final conversation with the patient	No	No	Partially founded	Warning	
23	2014	Abdomen	Radiologist	236	Error in diagnosis	Yes	Yes	Unfounded		
24	2014	Musculoskeletal	Radiologist	171	Failure to check INR, failure to perform angiography, and persisting in wait-and-see policy	Yes	Yes	Unfounded		
25	2014	Neuro	Radiologist	244	Forgery	No	Yes	Unfounded		Yes, rejected
26	2015	Musculoskeletal^#^	Radiologist	85	Error in diagnosis	No	Yes	Unfounded		Yes, rejected
27	2015	Musculoskeletal^#^	Radiologist	85	Error in diagnosis	No	Yes	Unfounded		Yes, rejected
28	2015	Musculoskeletal^#^	Radiologist	85	Error in diagnosis	No	Yes	Unfounded		Yes, rejected
29	2016	Musculoskeletal	Radiologist	399	Breach of doctor-patient confidentiality	No	Yes	Unfounded		
30	2016	Interventional	Radiologist	163	*Failure to obtain informed consent*, treatment error, and leaving the patient alone after the procedure	Yes	Yes	Partially founded	Warning	
31	2016	Vascular	Radiologist	325	Failure to propose urgent CT	No	Yes	Unfounded		
32	2016	Chest	Radiologist	181	Failure to adequately communicate a critical finding to the referring physician	Yes	Yes	Unfounded		
33	2017	Interventional	Radiologist	224	Lack of informed consent, treatment error, and providing insufficient information about the procedure	Yes	Yes	Unfounded		Yes, rejected
34	2017	Interventional	Radiologist	385	Part of the treatment performed by an inexperienced resident, treatment error, and *treatment delay after occurrence of complication*	No	Yes	Partially founded	Warning	Yes, granted
35	2017	Neuro	Radiologist	336	*Error in diagnosis*, failure to consult colleagues, failure to ask for external expertise, and failure to discuss with the referring physician and in the neuroradiology meeting	No	Yes	Partially founded	Warning	
36	2017	Abdomen	Radiologist	272	Failure to take medical history and physical examination, incomplete examination, and incorrect interpretation	No	Yes	Unfounded		
37	2017	Vascular	Resident	244	Failure to directly consult a vascular surgeon and failure to immediately hospitalize the patient.	No	Yes	Unfounded		
38	2017	Vascular	Radiologist	244	*Failure to directly consult a vascular surgeon and failure to immediately hospitalize the patient*	No	Yes	Founded	Warning	
39	2018	Musculoskeletal	Radiologist	336	Failing to determine preprocedural INR, carelessness in performing the procedure, insufficient aftercare, failure to ensure patient safety, incomplete and careless reporting, providing incomplete medical file, and breach of medical secrecy	Yes	Yes	Unfounded		
40	2018	Breast	Radiologist	151	Error in diagnosis	No	Yes	Unfounded		
41	2018	Breast	Radiologist	151	Error in diagnosis	No	Yes	Unfounded		
42	2018	Musculoskeletal	Radiologist	195	Not taking care of wheelchair transport for the patient	No	No	Unfounded		
43	2018	Musculoskeletal	Radiologist	168	*Error in diagnosis*, failure to recommend additional imaging, and *failure to add an addendum and to inform the referring physician after being aware of initial incorrect interpretation*	No	Yes	Partially founded	Warning	
44	2019	Neuro	Radiologist	196	Communication of erroneous preliminary findings and failure to communicate the results of the final report to the referring physician	No	No	Unfounded		
45	2019	Neuro	Radiologist	196	Communication of erroneous preliminary findings and failure to communicate the results of the final report to the referring physician	No	Yes	Unfounded		
46	2019	Interventional	Radiologist	277	Treatment error	No	Yes	Unfounded		
47	2019	Interventional	Radiologist	175	Insufficient preprocedural information, treatment error, and treatment delay after occurrence of complication^@^	No	Yes	Partially founded^@^	Warning	
48	2019	Head and neck	Radiologist	370	Purposefully withholding and manipulating medical data, and using these manipulated medical data in the radiology report	No	No	Unfounded		

*Same case

^#^Same case

^Allegations which were judged to be founded by the Dutch Medical Disciplinary Court at the initial verdict are italicized

^@^The allegations by the patient were judged to be unfounded. However, the Dutch Medical Disciplinary Court judged that the radiologist made an incorrect interpretation

**Table 3:** Potential causes leading to error and malpractice in 8 cases in which the allegation was judged (partially) founded by the Dutch Medical Disciplinary Court

Practical strategies to avoid error and malpractice^#^	Cases with (potential) failure
Practice within the limits of one’s expertise	
Use clinical information	
Obtain informed consent for invasive procedures	- Case 30 (failure to obtain informed consent before bronchial artery embolization procedure, which was complicated by inadvertent embolization of a spinal artery)
Stick to search patterns and know blind spots	- Case 18 (missed skin invasion in breast cancer) - Case 22 (missed retropharyngeal abscess) - Case 35 (missed cerebral peduncle infarction) - Case 43 (missed volar intercalated segmental instability) - Case 47 (missed contrast extravasation after endovascular treatment of popliteal artery occlusion)
Diligently review the entire study	
Double check known problem areas	
Avoid heuristics (particularly satisfaction of search, bias from context or prevalence, and anchoring to provided information)	
Be wary of inattentional blindness	
Do not rush a difficult case if is not overly time sensitive	
Use differential diagnosis	- Case 5 (breast carcinoma interpreted as lipoma)
Consult liberally with colleagues, especially in case of doubt	- May apply to all cases
The report should be clear and concise	
If colleagues were consulted, reference that in the report	
Recommend appropriate follow-up studies or recommendations	- Case 38 (failure to provide immediate care for a patient with pending rupture of a large iliac artery aneurysm)
Use disclaimers where appropriate	
Proofread reports	- Case 5 (incorrectness in the report: incorrectly stating that patient refused to undergo imaging)
Communication needs to be timely, appropriate, and documented	- Case 38 (failure to immediately consult a vascular surgeon for a patient with pending rupture of an iliac artery aneurysm) - Case 43 (failure to add an addendum and to inform the referring physician after being aware of initially missed volar intercalated segmental instability)

In two cases (cases 6 and 34), the appeal against the initial verdict was granted and the previously imposed disciplinary measures were reversed; these two cases are not included in this table

^#^Largely adopted from references [20] and [21]

## Discussion

The results of our study show that the Dutch Medical Disciplinary Court handles a mean of 4.8 allegations against radiologists related to radiology practice per year, and that this frequency has remained stable over the past 10 years. A mean of 4.8 allegations per year can be considered few, given that the mean number of cases against all Dutch health care professionals is 1709 per year [22] and that there are nearly as much as 1300 regular registered radiologists in The Netherlands at present [23]. There is no real financial obstacle or risk for a patient to file an allegation against a health care professional at the Dutch Medical Disciplinary Court. Patients can file an allegation for a total amount of 50 €, which will be refunded if the allegation is judged to be (partially) founded [24]. This very much contrasts with the civil court in The Netherlands, where the costs of the lawsuit process and the legal fees of the winning party have to be paid by the losing party if decided by the judge [25]. The relatively low number of allegations against radiologists filed at the Dutch Medical Disciplinary Court may be explained because there is not a real compensation culture in The Netherlands yet. One may also speculate that individual healthcare institutions handle a lot of patient complaints by themselves, which could reduce or avoid the number of allegations filed at the Dutch Medical Disciplinary Court. However, written complaints regarding radiological procedures in The Netherlands are also relatively few (14.4 per 100,000 radiological procedures) [26]. Therefore, the relatively low number of allegations may also indicate an overall high quality of radiology practice in The Netherlands. Accordingly, The Netherlands is frequently ranked as having one of the best healthcare systems in Europe [27].

A minority of allegations were judged to be (partially) founded. The Dutch Medical Disciplinary Court imposed 8 warnings and 2 reprimands to radiologists in the past 10 years (of which two were rejected after appeal). These disciplinary measures are the lowest penalties which can be imposed by the Dutch Medical Disciplinary Court. However, the impact of the disciplinary process and the measures itself should not be underestimated. Alleged healthcare professionals describe feelings of misery and insecurity both during the process as in its aftermath, and they fear receiving new complaints and provide care more cautiously after the imposed measure [28, 29]. This in turn may lead to defensive medicine, which is an important contributor to healthcare costs without adding any benefit to patients [30–33].

Error in diagnosis was the most common allegation (39.6%) filed at the Dutch Medical Disciplinary Court and most allegations were in the subspecialties breast imaging and musculoskeletal radiology. These findings are in accordance with previous studies on medical malpractice suits in the USA [1, 34], the UK [6], and Italy [7]. Errors are common, with an estimated day-to-day rate of 3–5% of radiology studies reported [35]. Radiologist reporting performance cannot be perfect, and some errors are inevitable [35]. However, there are strategies to avoid error and malpractice and we can learn from our mistakes (see Table 3). We also refer to the informative medicolegal series by L. Berlin, which have been published in the *American Journal of Roentgenology* in the past years [36]. We further note that radiologists should think about the consequences of error and malpractice in the context of the trend of using artificial intelligence. However, the question of “who is responsible for the diagnosis” when using artificial intelligence (being it either data scientists, manufacturers, and/or radiologists) remains to be answered [37].

Our study has some limitations. First, because our study included only data from The Netherlands, it is not sure whether our results are generalizable to other (European) countries, which have different law systems. Notably, a study which was published in 2010 showed a much higher risk of medical malpractice litigation for Italian radiologists, which was comparable to that for radiologists in the USA [7].

Italy, however, may be an exception among European countries [38]. Second, we only included data from the Dutch Medical Disciplinary Court. Because patients may also proceed to the civil court where they can file an allegation in parallel or separately from the Dutch Medical Disciplinary Court, the number of all official allegations may be underestimated. However, it was not possible to perform an unbiased research of civil court data, because only a selected part of civil court verdicts are publicly published [39]. Furthermore, the Dutch Medical Disciplinary Court essentially differs from civil court in that its main objective is to maintain and improve the quality of healthcare rather than punishing healthcare professionals. Third, we did not investigate the amount of time and attorney costs (83.3% used an attorney during the court session) spent by defendants. Fourth, we did not investigate the psychological impact of disciplinary measures on radiologists and whether these disciplinary measures achieved their primary goal: to maintain and improve the quality of healthcare. The systematic presentation of cases in this article may further contribute to the quality of radiology practice in general.

In conclusion, allegations against radiologist at the Dutch Medical Disciplinary Court are relatively few, their number has remained stable over the past 10 years, and a minority were judged to be (partially) founded. We can learn from the cases presented in this article, which may improve patient care.

## Abbreviations

UK: United Kingdom
USA: United States of America
